# Porous Phantoms Mimicking Tissues—Investigation of Optical Parameters Stability Over Time

**DOI:** 10.3390/ma14020423

**Published:** 2021-01-16

**Authors:** Paulina Listewnik, Monika Ronowska, Michał Wąsowicz, Valery V. Tuchin, Małgorzata Szczerska

**Affiliations:** 1 Department of Metrology and Optoelectronics, Faculty of Electronics, Telecommunications and Informatics, Gdańsk University of Technology, Narutowicza Street 11/12, 80-233 Gdańsk, Poland; pauliste@student.pg.edu.pl; 2 Biophoton, Gdańsk University of Technology, Narutowicza Street 11/12, 80-233 Gdańsk, Poland; mronowska@gmail.com; 3 Department of Morphological Sciences, Faculty of Veterinary Medicine, Warsaw University of Life Sciences, 02-776 Warszawa, Poland; michal_wasowicz@sggw.pl; 4 Research-Educational Institute of Optics and Biophotonics, Saratov State University, Astrakhanskaya 83, Saratov 410012, Russia; tuchinvv@mail.ru; 5 Laboratory of Laser Diagnostics of Technical and Living Systems, Institute of Precision Mechanics and Control RAS, Rabochaya 24, Saratov 410028, Russia; 6 Interdisciplinary Laboratory of Biophotonics, National Research Tomsk State University, Lenin’s Av. 36, Tomsk 634050, Russia

**Keywords:** absorption, coefficient stability, optical parameters, optical phantoms, phantoms mimicking tissue

## Abstract

Optical phantoms are used to validate optical measurement methods. The stability of their optical parameters over time allows them to be used and stored over long-term periods, while maintaining their optical parameters. The aim of the presented research was to investigate the stability of fabricated porous phantoms, which can be used as a lung phantom in optical system. Measurements were performed in multiple series with an interval of 6 months, recreating the same conditions and using the same measuring system consisting of an integrating sphere, a coherent light source with a wavelength of 635 nm and a detector. Scattering and absorption parameters were determined on the basis of the measured reflectance and transmittance. The tested samples were made of silicone and glycerol in various proportions.

## 1. Introduction

Due to constant development of new technological directions in the field of physics, engineering, medicine and in the era of changing trends, instead of using live tissues, optical phantoms have been used. Phantoms are made to reduce the need of natural biological tissues by replacing them with objects that mimic their optical properties. They are often used for non-invasive medical diagnostics, laser therapy and diagnosis of skin cancer [1,2]. Phantoms exhibit optical parameters of tissues; hence they are used in research to imitate the distribution of light in live tissue, including organs with an internal vascular system [3,4,5]. They are developed and used for several purposes:

- recording reference measurement using optical measuring devices and techniques [6,7]
- calibration of optical devices
- planning the distribution of light using physical tissue geometry [6,8].

Currently, optical techniques are becoming more and more popular as non-invasive tools for medical diagnostics. In addition to the basic parameters, such as absorption and scattering, phantoms also have other practical properties. Among them we can distinguish homogeneity, flexibility, durability, low cost, short production time, ease of production and possibility to create complex systems with internal heterogeneities, which include microchannels or capillaries [9].

In practice, two types of phantoms are discerned depending on the way the light is scattered inside of the material. These include internal scattering and scattering induced by nanoparticles or microparticles [10,11].

In nanoparticles or microparticles based phantoms, obtaining the equivalent of the scattering coefficient is controlled by the size, shape, concentration and type of nanoparticles, such as alumina [12], polymer microspheres [6,13], titanium dioxide, zinc oxide [14,15,16,17].

The matrices for the above particles are polyurethane and polyester resin [18], polyvinyl chloride plastisol [19], and silicone [12].

The resulting systems are not quite an integral system due to the uncontrolled heterogeneity of phantom properties, resulting from sedimentation or uneven concentration of nanoparticles in the matrix space. Such features affect the uneven distribution of the scattering centers. Opportunities affecting the resolution of these obstacles require additional equipment, thus increasing the cost, scale of difficulty and the time of production of the finished product in the form of a phantom. There are also particle-free forms with internal scattering. They are an ideal example of phantoms due to their homogeneity and easier production method. Phantoms having internal homogeneous dispersion were created based on agarose, fibrin, or collagen matrix for encapsulation of the dispersed Intralipid solution [6,20,21,22]. Particle-free phantoms are also remarkable in that they can serve as models for porous biological tissues such as brain and lung [23,24,25,26]. For brain, pores size of extracellular space medium is of approximately 1–4 μm [25]. The lung’s internal structure within the respiratory parenchyma shows a gas exchange surface that is divided into a large number of small subunits (alveoli) connected to a branched conducting airway system. The mean alveolar number in humans is of 274–790 million with the mean size of a single alveolus of 4.2 × 10^6^ μm^3^ or approximately 200 μm in diameter. Air-filled lungs present significant challenges for optical imaging including optical coherence tomography (OCT) because of the large refractive-index mismatch between alveoli walls and the enclosed air-filled region [23]. During OCT imaging, the light is strongly backscattered at each air-tissue interface, such that image reconstruction is typically limited to a single alveolus.

At the same time, the filling of these cavities with an optical clearing agent (OCA), to which water (physiological solution) can also be attributed, since its refractive index is much higher than that of air will lead to much better tissue optical transmittance. For the first time, this has been proven in [23] by using freshly excised, intact lungs from two sheep and the experimental setup with the optofluidic OCT needle probe when lung areas perfused with saline show a marked improvement in image quality and image penetration depth. 

The purpose of this study is to assess stability of optical parameters and to investigate optical clearing of the porous phantoms, which is crucial during validation of optical measurement methods and long-term measurements.

## 2. Materials and Methods

**Figure 1:**
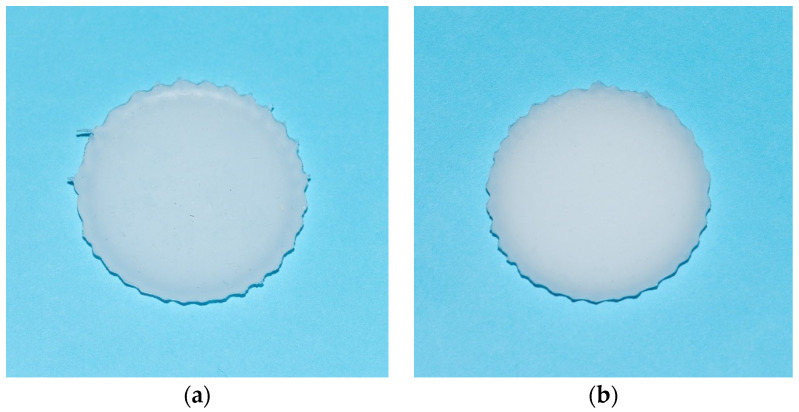
Investigated optical phantoms, where: (**a**) sample with 5 mL of glycerol, (**b**) sample with 2 mL of glycerol.

This study was conducted on optical phantoms produced on a basis of polydimethylsiloxane (PDMS, Sylgard^®^ 184, Dow Corning, Midland, MI, USA) and glycerol, shown in Figure 1.

The PDMS is a component of organic silicone and curing agent which solidifies at room temperature over the period of 48 h. After curing, it is transparent in the visible-near-infrared (VIS-NIR) range, non-toxic, non-flammable, stabling, and hydrophobic [6]. The samples composition differed in the amount of added glycerol. One includes 2 mL of glycerol, while the other contains 5 mL. The production process was described in elsewhere [6].

**Figure 2:**
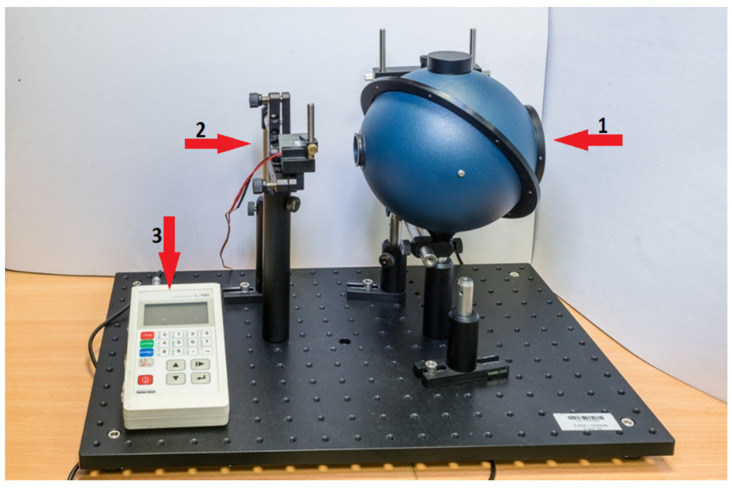
The experimental setup utilized in this study, where: 1—integrating sphere, 2—light source, 3—luxmeter.

The measurement system, shown in Figure 2, consisted of an integrating sphere 4P-GPS-053-SL (Labsphere Inc., North Sutton, NH, USA) with a Spektralon^®^ coating. This type of coating characterized by high diffuse reflectance (over the 250–2500 nm spectral range) with reflectance more than 99% for a wavelength range between 400 and 1500 nm. A laser diode module with a wavelength of 635 nm (red) was used as a light source and it operated with an optical power of 3 mW, while Luxmeter L-100 with a dedicated measuring head (Sonopan, Białystok, Poland) was used as a detector. 

Optical parameters of the phantoms were examined in a series of measurements. The measurements were repeated six months later using the exact same samples and experimental setup. The scattering and absorption parameters were determined based on the measured reflectance and transmittance. 

**Figure 3:**
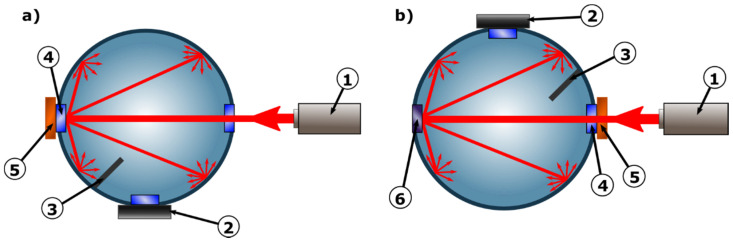
Principle of operation of a measurement system, (**a**) in reflectance mode and (**b**) in diffuse transmittance mode, where: 1—light source, 2—luxmeter, 3—baffle, 4—sample port, 5—optical phantom, 6—unused plugged port [27].

The integrating sphere, light source and detector have been precisely mounted to the optical breadboard (Thorlabs, Newton, NJ, USA), therefore enabling to construct a stable system, which provides repeatability and accuracy of measurements. To simplify calculations, only one integrating sphere was used in this measurement system. This was further possible thanks to the mechanical design, allowing the sphere to rotate around its axis. The design of the sphere itself, and more precisely the baffle located between the 0 degrees and 90 degrees ports enabled the detector to be permanently installed in the 90 degrees port, as is presented in Figure 3.

In the described setup it was possible to take measurements of both transmittance and reflectance with the light source and detector in a fixed placement. Depending on the measurement mode, the sphere was rotated by exactly 180 degrees. To limit inaccuracy of measurements all external factors that could affect the readings were eliminated, laser was properly stabilized and a series of calibration measurements was performed.

To demonstrate designed phantoms applicability for modeling of lung tissue and testing of different OCAs, collimated transmittance spectral measurements were conducted using phantom samples with the initial thickness of 0.5 mm (Figure 1). Each sample was fixed on a wire frame which was inserted into a glass cuvette with an OCA. A cuvette with a sample was placed between two QP400-1-UV-VIS optical fibers (Ocean Optics, Orlando, FL, USA) with a core diameter of 400 μm and 74-ACR collimators (Ocean Optics, USA). Halogen lamp HL-2000 (Ocean Optics, USA) was used as a light source. All measurements were carried out at room temperature (about 25 °C). The collimated transmittance spectra of the skin were recorded every 30 or 60 s for up to 205 min of immersion using a USB4000-Vis-NIR spectrometer (Ocean Optics, USA) in the wavelength range of 600–900 nm. 

The collimated transmittance of the sample $T_{c}(t)$ over time can be written as [28]:

(1) $$T_{c}\left(t\right)\propto 1-exp\left(-\frac{t}{\tau }\right),$$

where:

(2) $$\tau =\frac{l^{2}}{\pi^{2}D_{a}},$$

where: τ is the characteristic time of an OCA diffusion at its delivery through both sample surfaces; *l* is the sample thickness, which is much less than its area, and $D_{a}$ is the effective diffusion coefficient of the OCA. The diffusion of OCA molecules in phantoms (tissues) can be considered as a process hindered by a complex material structure, which leads to an increase in molecular path length caused by their interaction with these obstacles. This hindered mobility in phantoms (tissues) relative to free medium (water or very diluted gel) is quantitatively introduced as the tortuosity [25,26,29,30,31,32]:

(3) $$\frac{l_{d}}{L}=\sqrt{\frac{D_{a}^{\mathrm{free}}}{D_{a}}},$$

where: $D_{a}^{\mathrm{free}}$ is the diffusion coefficient of an OCA in a free medium and $D_{a}$ is the effective diffusion coefficient accounting for elongation of diffusion path. Tortuosity is a measure of the geometric complexity of a porous medium, such as phantom (tissue), and can be introduced as the ratio of the path length of the molecular flow between two points *l_d_* to the direct distance between these points *L*.

From Equation (1) the OC efficiency (OCE), which is defined as a ratio of collimated transmittance after OC $T_{c}^{\mathrm{OC}}$ to its initial value $T_{c}^{0}$, can be introduced:

(4) $$\mathrm{OCE}=\frac{T_{c}^{\mathrm{OC}}}{T_{c}^{0}}.$$

The transmission of a narrow collimated light beam through a sample thin layer of thickness *l* is described by Bouguer−Beer−Lambert law [28]:

(5) $$T_{c}=\frac{I\left(l\right)}{I_{0}}=\exp\left(-\mu_{t}l\right),$$

where: *I*_0_ and *I*(*l*) are the intensities of the incident and transmitted light, respectively;

(6) $$\mu_{t}=\mu_{a}+\mu_{s},$$

is the light attenuation (extinction) coefficient, *μ_a_* is the absorption coefficient, and *μ_s_* is the scattering coefficient. For the soft tissues in the visible and near-infrared ranges, $\mu_{s}\gg \mu_{a}$, the same is for designed phantoms.

For many soft tissues, composed of Mie scatterers, for which scattering anisotropy factor *g* ≥ 0.9, diameter 2*a* and relative refractive index $m=n_{s}/n_{0}$ are in the range 5 < (2π*a*/*λ*) < 50 and 1.0 < *m* <1.1, respectively, reduced scattering coefficient is described as [28,33]

(7) $$\mu_{s}^{\prime }=3.28\pi a^{2}\rho_{s}{\left(\frac{2\pi n_{0}a}{\lambda_{0}}\right)}^{0.37}{\left(m-1\right)}^{2.09},$$

(8) $$\mu_{s}^{\prime }=\left(1-g\right)\mu_{s},$$

where: *ρ*_s_ is the volume density of the scatterers, *λ*_0_ is the wavelength of the incident light, $n_{s}$ and $n_{0}$ are the refractive indices of the scatterers and the background medium, respectively. 

For porous media, for which *m* < 1.0, these equations are also applicable [28]. It follows that for refractive index matching of the scatterers $n_{s}$ and the background medium $n_{0}$, i.e., m→1, both scattering $\mu_{s}$ and reduced scattering $\mu_{s}^{\prime }$ coefficients go down. Supposing that at immersion of the sample in an OCA diameter 2*a* and density *ρ_s_* of scatterers (partially air-filled cavities) do not changed and only relative index of refraction $m=n_{s}/n_{0}$ is changed with time *t*, from Equation (7) it is easy to get expression for scattering coefficient time dependence $\mu_{s}^{\mathrm{OC}}\left(t\right)$ as a function of relative refraction index $m^{\mathrm{OC}}\left(t\right)$ in the course of OC:

(9) $$\mu_{s}^{\mathrm{OC}}\left(t\right)≅\left[\mu_{s}\left(t=0\right)\right]\times {\left(\frac{m^{\mathrm{OC}}\left(t\right)-1}{m\left(t=0\right)-1}\right)}^{2},$$

This expression can be used to evaluate OC efficiency (OCE) using Equations (4)–(6). Refractive index of lung mucosal tissue *n*_0_ = *n*_lung_ = 1.37 [28], thus for alveoli filled up by the air *n*_s_ = *n*_air_ = 1, we have *m*(*t* = 0) = 0.73. At OC by filling up alveoli with water *n*_s_ = *n*_w_ = 1.33 we have $m^{\mathrm{OC}}\left(t\right)$ = 0.97. Thus at OC $\mu_{s}^{\mathrm{OC}}\left(t\right)≅0.01\times \left[\mu_{s}\left(t=0\right)\right]$, i.e., scattering coefficient of lungs can be significantly decreased, up to 100 times. Indeed, this is an overestimation, because it is difficult to expect that all alveoli will be filled up fully by water. Therefore, experimental data for the appropriate phantom should be of great importance in testing of different OCAs.

## 3. Results

### 3.1. Optical Parameters Measurements

**Figure 4:**
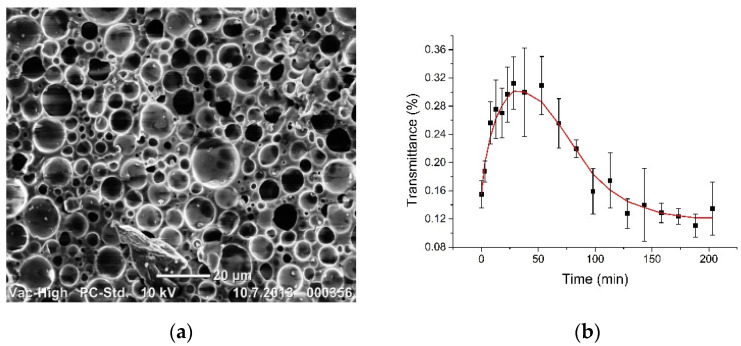
(**a**) SEM of the internal structure of the nanoparticle-free silicone-glycerol phantom; (**b**) the kinetics of collimated transmittance averaged for spectral band 600–800 nm and presented as (mean ± SD) at impregnation of the sample by distilled water (**b**).

In Figure 4a, there is presented the SEM of the internal structure of the silicone-glycerol phantom [6], where cavities with mean diameter of ~4 µm size and partially filled up by air are well seen. Normally they provide its strong scattering and white colour appearance as it well seen in Figure 1.

Figure 4b shows our experimental studies for collimated transmittance of phantoms, which demonstrate well the possibility of optical clearing of porous medium using water and therefore confirm the possibility of lung tissue modeling and testing of OCAs. The initial thickness is 0.5 mm and weight of the sample was (0.10 ± 0.01) g and after 205 min kept in a water bath it was swelled with the increased weight up to (0.20 ± 0.01) g, but the turbidity is similar to that was at the beginning. For water, the optical clearing efficiency OCE (Equation (4)) is estimated as (1.9 ± 0.5)-fold with the characteristic time of approximately 12.5 min, determined by fitting experimental kinetic curve in Figure 4b with Equation (1) in the time range from t = 0 to 25 min, where it is valid. For longer impregnation time collimated transmittance was saturated and went back to the initial state, which can be explained by sample swelling and relatively low refractive index of water. The similar data was found for freshly excised sheep lungs at their impregnation by saline and measurements using optofluidic OCT needle probe, where optical penetration depth *l*_t_ ≅ 1/μ_s_ was changed from approx. 150 μm to 300 μm [23].

**Figure 5:**
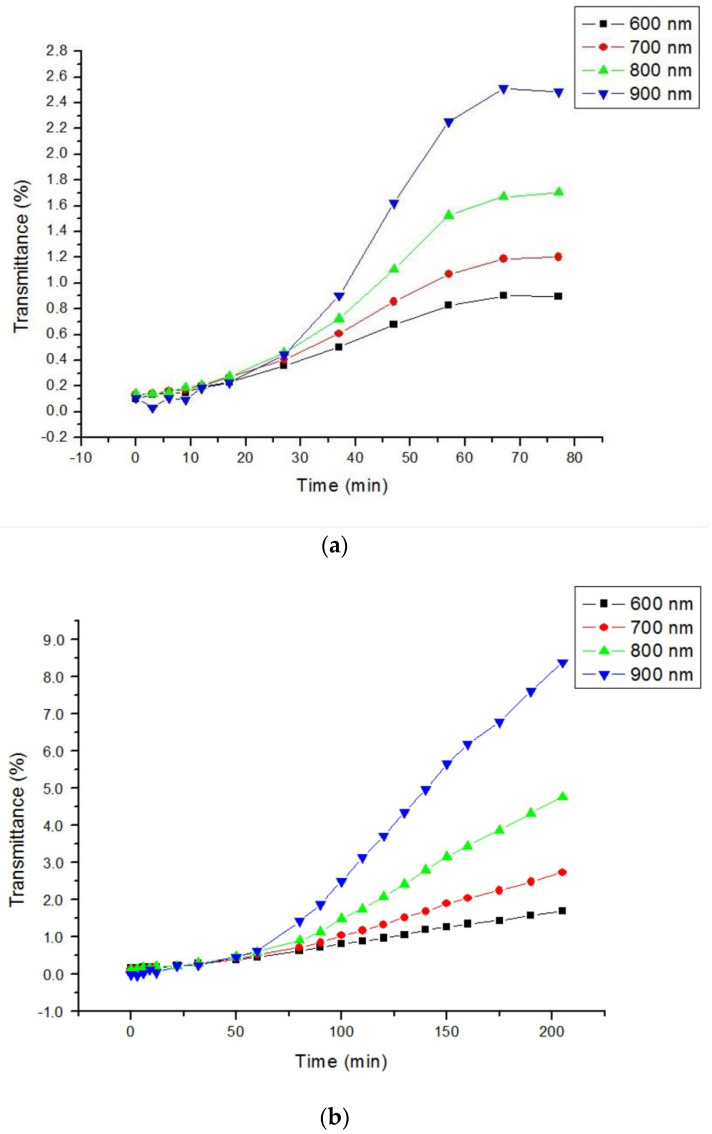
The kinetics of collimated transmittance at impregnation of the sample by: (**a**) aqueous 40% glucose solution; (**b**) at impregnation of the sample by aqueous 50% DMSO solution. All experimental errors expressed as SD were less than the size of point icons.

For two other tested OCAs, such as 40% glucose and 50% DMSO with higher refractive indices, OCE is much higher and saturated at much longer soaking times (Figure 5). In particular, for 40% glucose OCE = 26 fold with τ = 50 min and for 50% DMSO OCE = 85 fold with τ = 200 min at 900 nm. The high efficiency of DMSO is associated with its high permeability to various materials. For glucose solution, the initial weight of the sample was (0.14 ± 0.01) g and after 76 min kept in solution it was swelled with the increased weight up to (0.20 ± 0.01) g, while for DMSO solution the initial weight of the sample was (0.12 ± 0.01) g and after 205 min kept in solution it was swelled with the increased weight up to (0.16 ± 0.01) g. In both cases, the turbidity is significantly less than it was at the beginning. 

### 3.2. Optical Parameters Stability Measurements

The values obtained from executed measurements were used to calculate the diffuse reflectance Rd and the diffuse transmittance Td, using the Formulas (10) and (11):

(10) $$R_{d}=\frac{P_{R\;sample}}{P_{\;reference}},$$

(11) $$T_{d}=\frac{P_{T\;sample}}{P{\;}_{reference}}$$

where: *P_Rsample_*—the detected signal of the investigated phantom measured in the reflectance mode, *P_reference_*—the detected signal obtained from measurement of a calibration plate, *P_Tsample_*—the detected signal of the investigated phantom measured in the transmittance mode.

The values obtained from measurements allowed to determine the absorption coefficient *µ_a_* and the reduced scattering coefficient *µ_s_′* based on Kubelka–Munk model, according to the formulas presented below [34]:

(12) $$S_{KM}=\frac{1}{d\sqrt{a^{2}-1}}ln\left[\frac{1-R_{d}\left(a-\sqrt{a^{2}-1}\right)}{T_{\;d}}\right],$$

(13) $$A_{KM}=\;\left(a-1\right)S_{KM},$$

(14) $${µ}_{s}^{\prime }=\;\frac{4}{3}S_{KM}+\frac{1}{6}A_{KM},$$

(15) $${µ}_{a}=\;\frac{1}{2}A_{KM},$$

where: *S_KM_* –Kubelka–Munk scattering coefficient, *A_KM_*—Kubelka–Munk absorption coefficient, *d*—sample thickness and:

$$a=\;\frac{1-T_{d}^{2}+R_{d}^{2}}{2R_{d}},\;\;b=\;\sqrt{a^{2}-1}.$$

The stability of the investigated phantoms was calculated from the following formula:

(16) $$\delta =\left|\frac{\upsilon_{M}-\upsilon_{R}}{\upsilon_{R}}\right|\times 100\%,$$

where: *δ*—stability, *υ_M_*—measured value, *υ_R_*—reference value.

**Figure 6:**
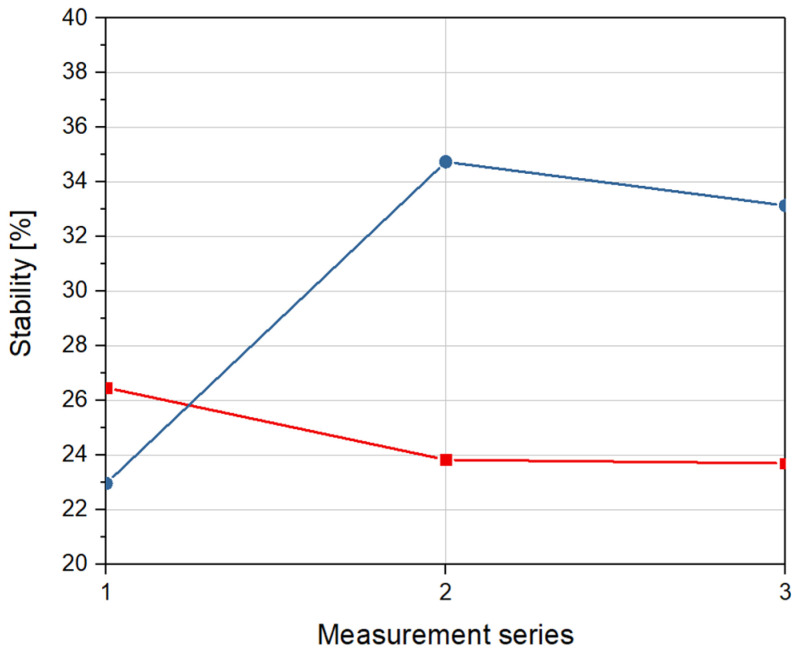
Stability of absorption coefficient for a sample with 2 mL of glycerol (red) and a sample with 5 mL of glycerol (blue) over time, in relation to reference measurement.

Figure 6 presents stability of absorption parameters of optical phantoms. 

Each measurement series represents the stability of absorption coefficients of investigated optical phantoms in relation to reference data. As shown in Figure 6, the stability of phantoms with 5 mL of glycerol are almost 1.5 times bigger than the stability of phantoms with 2 mL of glycerol. The maximum calculated stability of absorption coefficients for both samples equals 34.7%. 

**Figure 7:**
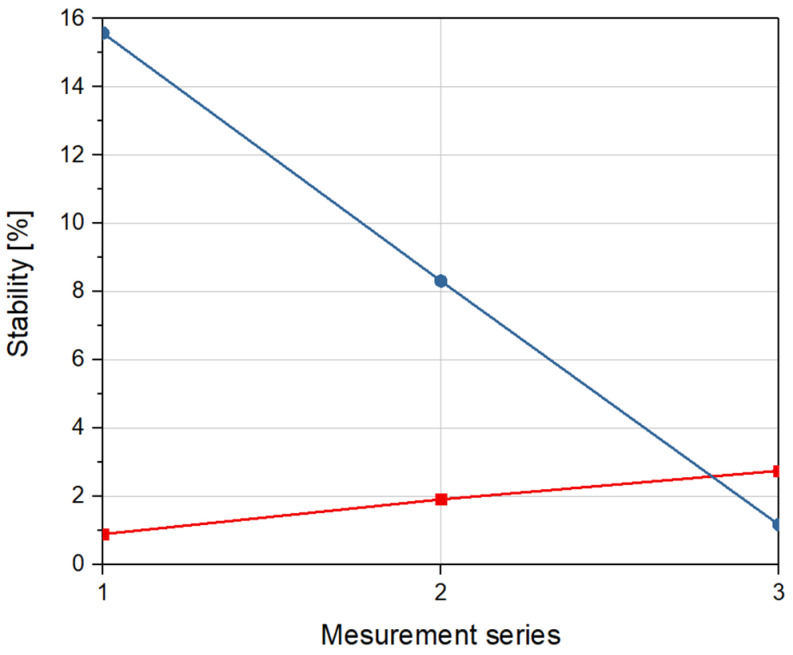
Stability of scattering coefficient for a sample with 2 mL of glycerol (red) and a sample with 5 mL of glycerol (blue) over time, in relation to reference measurement.

Stability of scattering parameters of optical phantoms is shown in Figure 7. Each measurement series represents the stability of scattering coefficients of investigated optical phantoms in relation to reference data.

As can be seen in Figure 7, unlike the absorption parameters, the stability of phantoms with 5 mL of glycerol are significantly lower than the stability of phantoms with 2 mL of glycerol. The maximum calculated stability of scattering coefficients for both samples equals 15.6%. 

## 4. Conclusions

The presented values of the optical parameters of the phantoms, such as the scattering coefficient and the absorption coefficient, were obtained by performing a series of measurements with an interval of 6 months. The maximum stability of the absorption coefficient for the sample with 2 mL of glycerol was 26.5% and for the sample with 5 mL of glycerol it was 34.7%, with respect to reference data. The values of stability of scattering coefficient were 2.8 and 15.6%, respectively. It was shown that at the wavelength of 635 nm, the investigated optical parameters of phantoms are preserved. It also means that they can be stored for a long time as they are not biodegradable. Phantoms are widely used in the calibration and validation of optical measurement methods. The stability of their optical parameters makes it possible to use them repeatably. In addition, it could be checked whether it is stable with other light sources.

Designed phantoms can serve for mimicking of optical properties of the specific porous tissues such as brain and lung, for testing of optical clearing agents and evaluation of tissue perfusion by metabolic fluids. 

## Data Availability

Data available in a publicly accessible repository.

## References

[1] Feder I., Duadi H., Fixler D. (2015). Experimental System for Measuring the Full Scattering Profile of Circular Phantoms. Biomed. Opt. Express.

[2] Duadi H., Feder I., Fixler D. (2014). Linear Dependency of Full Scattering Profile Isobaric Point on Tissue Diameter. J. Biomed. Opt..

[3] Jacques S.L. (2013). Optical Properties of Biological Tissues: A Review. Phys. Med. Biol..

[4] Lu G., Fei B. (2014). Medical Hyperspectral Imaging: A Review. J. Biomed. Opt..

[5] Feder I., Wróbel M., Duadi H., Jędrzejewska-Szczerska M., Fixler D. (2016). Experimental Results of Full Scattering Profile from Finger Tissue-like Phantom. Biomed. Opt. Express.

[6] Wróbel M.S., Popov A.P., Bykov A.V., Tuchin V.V., Jędrzejewska-Szczerska M. (2016). Nanoparticle-Free Tissue-Mimicking Phantoms with Intrinsic Scattering. Biomed. Opt. Express.

[7] Karpienko K., Gnyba M., Milewska D., Wróbel M.S., Jędrzejewska-Szczerska M. (2016). Blood Equivalent Phantom vs Whole Human Blood, a Comparative Study. J. Innov. Opt. Health Sci..

[8] Ziemczonok M., Kuś A., Wasylczyk P., Kujawińska M. (2019). 3D-Printed Biological Cell Phantom for Testing 3D Quantitative Phase Imaging Systems. Sci. Rep..

[9] Iliescu C., Taylor H., Avram M., Miao J., Franssila S. (2012). A Practical Guide for the Fabrication of Microfluidic Devices Using Glass and Silicon. Biomicrofluidics.

[10] Delfino I., Lepore M., Esposito R. (2020). Optical Characterization of Homogeneous and Heterogeneous Intralipid-Based Samples. Appl. Sci..

[11] Ruiz C.C., Molina-Bolívar J.A., Aguiar J., MacIsaac G., Moroze S., Palepu R. (2001). Thermodynamic and Structural Studies of Triton X-100 Micelles in Ethylene Glycol–Water Mixed Solvents. Langmuir.

[12] Pogue B.W., Patterson M.S. (2006). Review of Tissue Simulating Phantoms for Optical Spectroscopy, Imaging and Dosimetry. J. Biomed. Opt..

[13] Chang R.C., Johnson P., Stafford C.M., Hwang J. (2012). Fabrication and Characterization of a Multilayered Optical Tissue Model with Embedded Scattering Microspheres in Polymeric Materials. Biomed. Opt. Express BOE.

[14] Kennedy B.F., Curatolo A., Hillman T.R., Saunders C.M., Sampson D.D. (2011). Speckle Reduction in Optical Coherence Tomography Images Using Tissue Viscoelasticity. J. Biomed. Opt..

[15] de Bruin D.M., Bremmer R.H., Kodach V.M., de Kinkelder R., van Marle J., van Leeuwen T.G., Faber D.J. (2010). Optical Phantoms of Varying Geometry Based on Thin Building Blocks with Controlled Optical Properties. J. Biomed. Opt..

[16] Liang X., Oldenburg A.L., Crecea V., Chaney E.J., Boppart S.A. (2008). Optical Micro-Scale Mapping of Dynamic Biomechanical Tissue Properties. Opt. Express.

[17] Grimwood A., Garcia L., Bamber J., Holmes J., Woolliams P., Tomlins P., Pankhurst Q.A. (2010). Elastographic Contrast Generation in Optical Coherence Tomography from a Localized Shear Stress. Phys. Med. Biol..

[18] Friebel M., Roggan A., Müller G., Meinke M. (2006). Determination of Optical Properties of Human Blood in the Spectral Range 250 to 1100 Nm Using Monte Carlo Simulations with Hematocrit-Dependent Effective Scattering Phase Functions. J. Biomed. Opt..

[19] Bohndiek S.E., Bodapati S., Van De Sompel D., Kothapalli S.-R., Gambhir S.S. (2013). Development and Application of Stable Phantoms for the Evaluation of Photoacoustic Imaging Instruments. PLoS ONE.

[20] Kennedy B.F., Loitsch S., McLaughlin R.A., Scolaro L., Rigby P., Sampson D.D. (2010). Fibrin Phantom for Use in Optical Coherence Tomography. J. Biomed. Opt..

[21] Fixler D., Nayhoz T., Ray K. (2014). Diffusion Reflection and Fluorescence Lifetime Imaging Microscopy Study of Fluorophore-Conjugated Gold Nanoparticles or Nanorods in Solid Phantoms. ACS Photonics.

[22] Ankri R., Taitelbaum H., Fixler D. (2011). Reflected Light Intensity Profile of Two-Layer Tissues: Phantom Experiments. J. Biomed. Opt..

[23] Quirk B.C., McLaughlin R.A., Pagnozzi A.M., Kennedy B.F., Noble P.B., Sampson D.D. (2014). Optofluidic Needle Probe Integrating Targeted Delivery of Fluid with Optical Coherence Tomography Imaging. Opt. Lett..

[24] Ochs M., Nyengaard J.R., Jung A., Knudsen L., Voigt M., Wahlers T., Richter J., Gundersen H.J.G. (2004). The Number of Alveoli in the Human Lung. Am. J. Respir. Crit. Care Med..

[25] Ray L.A., Heys J.J. (2019). Fluid Flow and Mass Transport in Brain Tissue. Fluids.

[26] Graczyk K.M., Matyka M. (2020). Predicting Porosity, Permeability, and Tortuosity of Porous Media from Images by Deep Learning. Sci. Rep..

[27] Listewnik P., Wąsowicz M., Kosowska M., Mazikowski A. (2019). A Measurement System for Quasi-Spectral Determination of Absorption and Scattering Parameters of Veterinary Tissue Phantoms. Appl. Sci..

[28] Tuchin V.V. (2015). Tissue Optics: Light Scattering Methods and Instruments for Medical Diagnosis.

[29] Hagberg G.E., Mamedov I., Power A., Beyerlein M., Merkle H., Kiselev V.G., Dhingra K., Kubìček V., Angelovski G., Logothetis N.K. (2014). Diffusion Properties of Conventional and Calcium-Sensitive MRI Contrast Agents in the Rat Cerebral Cortex: Effective Diffusion of MRI Contrast Agents. Contrast Media Mol. Imaging.

[30] Mériaux S., Conti A., Larrat B. (2018). Assessing Diffusion in the Extra-Cellular Space of Brain Tissue by Dynamic MRI Mapping of Contrast Agent Concentrations. Front. Phys..

[31] Conti A., Magnin R., Gerstenmayer M., Tsapis N., Dumont E., Tillement O., Lux F., Le Bihan D., Mériaux S., Della Penna S. (2019). Empirical and Theoretical Characterization of the Diffusion Process of Different Gadolinium-Based Nanoparticles within the Brain Tissue after Ultrasound-Induced Permeabilization of the Blood-Brain Barrier. Contrast Media Mol. Imaging.

[32] Jacques S.L., Pogue B.W. (2008). Tutorial on Diffuse Light Transport. J. Biomed. Opt..

[33] Graaff R., Aarnoudse J.G., Zijp J.R., Sloot P.M.A., de Mul F.F.M., Greve J., Koelink M.H. (1992). Reduced Light-Scattering Properties for Mixtures of Spherical Particles: A Simple Approximation Derived from Mie Calculations. Appl. Opt..

[34] Krainov A.D., Mokeeva A.M., Sergeeva E.A., Agrba P.D., Kirillin M.Y. (2013). Optical Properties of Mouse Biotissues and Their Optical Phantoms. Opt. Spectrosc..

